# Nattokinase-Associated Hemoperitoneum in an Elderly Woman

**DOI:** 10.7759/cureus.20074

**Published:** 2021-12-01

**Authors:** Lintu Ramachandran, Ammar Aqeel, Ali Jafri, Yadwinder Sidhu, Taha Mohamed Djirdeh

**Affiliations:** 1 Internal Medicine, Javon Bea Hospital, Rockford, USA

**Keywords:** over the counter supplement, herbal supplement adverse event, herbal thrombolytic, herbal supplements, nattokinase

## Abstract

The consumption of herbal supplements has become increasingly popular the United States. One such herbal supplement that is available at pharmacies and grocery stores is nattokinase. Nattokinase, a byproduct of soybean fermentation, may have some thrombolytic properties. We present the case of a patient who developed hemoperitoneum and subsequently passed away. We review the potential mechanisms of action of nattokinase and warn against consumption of herbal supplements, especially nattokinase.

## Introduction

Nattokinase, an enzyme produced by bacteria during soybean fermentation, has been shown to have some health benefits. While originally discovered in Japan, nattokinase has been gaining popularity in the United States and is currently available over the counter [1]. Nattokinase has been linked to improvement and prevention of cardiovascular diseases [2]. Nattokinase is a protease composed of 275 amino acids, which belongs to the subtilisin family and has high affinity for fibrin [3]. As such, there has been association between nattokinase and bleeding. We present the case of an elderly female with a history of atrial fibrillation, who experienced a bleeding complication, and passed away after taking over-the-counter nattokinase.

## Case presentation

**Figure 1:**
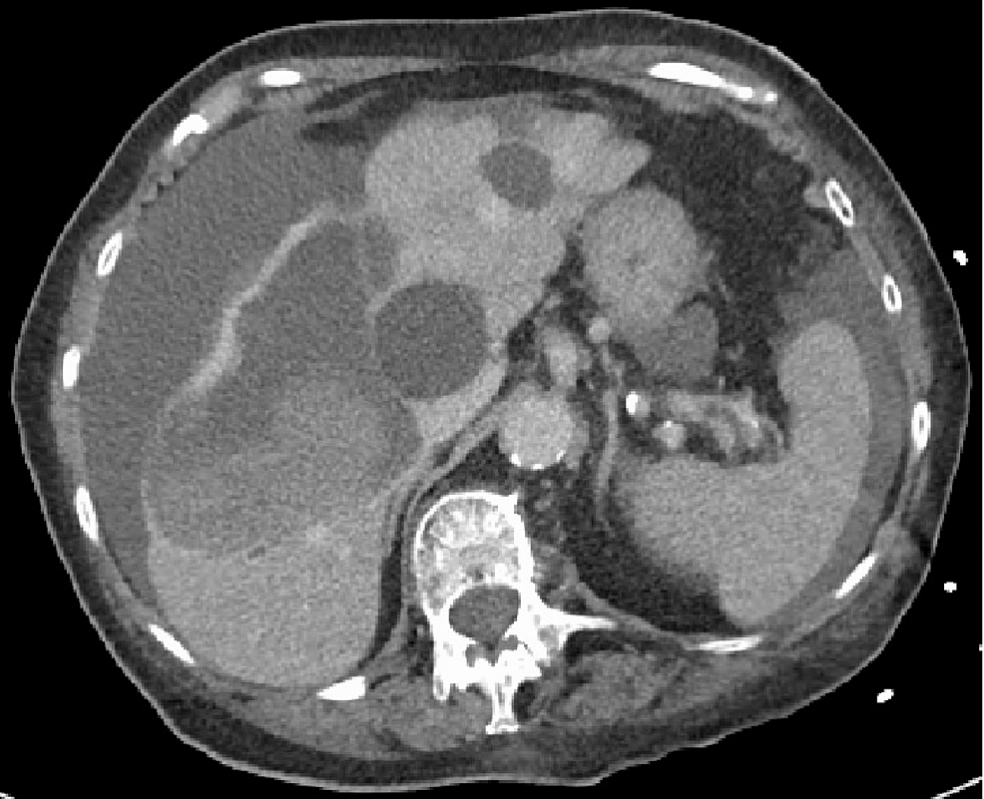
CT abdomen showing moderate hemoperitoneum along with multiple bleeding spots in the liver

Our patient is a 92-year-old female with a past medical history of chronic kidney disease, hypothyroidism, paroxysmal atrial fibrillation, and essential hypertension who presented to the hospital for a mechanical fall. She did not have any aura-like symptoms, chest pain, dizziness, vision loss, urinary incontinence, or fecal incontinence during, prior to, or after the event. She did not have any loss of consciousness at the time. Of note, our patient was not on any anticoagulants, but was taking nattokinase, a herbal supplement for her atrial fibrillation. She also denied aspirin use as well. On admission, her vitals were temperature of 36.8°C, heart rate of 77 beats per minute, respiratory rate of 16 breaths per minute, a blood pressure of 69/33 mmHg, and an oxygen saturation of 93% on 2 L via nasal canula. Physical examination showed a tired drowsy-appearing woman with mild abdominal tenderness to palpation diffusely. On laboratory evaluation she was found to have normocytic anemia with a hemoglobin of 4.28 mmol/L. Hematocrit was 26.4%. Platelet count was slightly decreased at 114,000/µL. International normalized ratio (INR) was 1.3. A fibrinogen level was not obtained. She was noted to have acute kidney injury with a creatinine of 194.5 µmol/L from a baseline creatinine of 141.5 µmol/L. Her sodium, potassium, chloride, magnesium, and bicarbonate levels were 143 mmol/L, 3.8 mmol/L, 107 mmol/L, 0.95 mmol/L, and 19 mmol/L, respectively. She received intravenous (IV) fluids and her blood pressure and mentation slightly improved. An urgent CT head was negative for acute bleeding. Focused assessment with sonography in trauma exam was positive for fluid in the abdomen. CT abdomen and pelvis showed ruptured hepatic cystic lesion with moderate hemoperitoneum as shown in Figure 1.

The patient did not have any prior CT abdomen to compare. The patient elected against surgery and preferred conservative management with transfusion and close monitoring, understanding the gravity of the situation. Her creatinine improved to 150.3 µmol/L after IV fluids. She received a total of 4 units of packed red blood cells (pRBCs) and 3 units of fresh frozen plasma. She continued to decompensate. The patient and her family declined surgical intervention, and elected for Do Not Resuscitate/Do Not Intubate. The patient passed away one week later from severe hypotension and bradycardia.

## Discussion

Nattokinase, an enzyme produced by the bacterium *Bacillus subtilis* during soybean fermentation, has been shown to have some thrombolytic properties. The history of nattokinase dates to 1987 when it was discovered and named by Sumi et al. [4]. Since then, there has been speculation about the cardiovascular benefits of nattokinase, with some studies reporting antihypertensive, lipid-lowering, and anticoagulating properties of nattokinase [5]. Recently, nattokinase has been gaining popularity as an over-the-counter thrombolytic.

Our patient had been ingesting nattokinase daily. She is unsure how many she takes, “sometimes a handful”. She lived alone and as such there was no one to confirm how many nattokinase supplements she had been taking daily. On admission, her INR was 1.3, prothrombin time (PT) was 13.3, and aPTT of 21.5. Her bleeding was rather difficult to stop, despite transfusion of 4 units of pRBCs throughout her hospital course. We hypothesize that at least part of this could be attributed to her intake of daily nattokinase. Several theories exist to explain how nattokinase acts as an anticoagulant. Jensen et al. showed that there was a 15% decrease in von Willebrand factor levels in female patients who took nattokinase for eight weeks versus females who took placebo [6]. Studies have also demonstrated an increase in activity of fibrinolysis and anticoagulant parameters between 2 and 8 hours after nattokinase intake [7].

The exact mechanism of fibrinolysis associated with nattokinase is an area of active ongoing research. Studies have shown increased PT and PTT levels in nondiabetic and hypercholesterolemic patients [8]. Increase in antithrombin levels in patients after the intake of nattokinase has been demonstrated. Antithrombin directly inhibit the levels of factors Xa and IXa, likely explaining the PT and aPTT levels seen in patients on nattokinase [9]. Our patient, however, did not have raised aPTT levels. Her labs revealed decreased PTT. There have been reported cases of nattokinase-associated bleeding in the setting of normal aPTT levels [10].

Antithrombin also directly inhibits thrombin (Factor IIa), which is likely another mechanism by which nattokinase intake can lead to coagulopathy without change in PTT levels, as in our patient. Another proposed mechanism of nattokinase is through degradation of fibrin and increasing plasmin levels [11]. Overall, further research needs to be conducted to examine the exact mechanism of fibrinolysis even further.

Nattokinase is sold as a dietary supplement in the United States, Canada, and Europe. The lack of regulation involved in dietary supplementation and lack of validated studies make nattokinase’s use unsafe and its efficacy questionable. Our patient ended up with severe bleeding and hemoperitoneum, ultimately dying from the complications that arose from the anticoagulative properties of nattokinase. The case highlights the need for further studies and regulation of nattokinase.

## Conclusions

In conclusion, while there have been numerous studies looking at the positive health benefits of nattokinase, we present a case that led to bleeding complication in our patient presumably from consuming nattokinase. The exact mechanism by which nattokinase leads to increased bleeding risk is unclear and is an area that would benefit from further research. We caution the use of herbal supplements such as nattokinase as the lack of FDA regulations and the potential for drug-drug interactions put patients at increased risk for complications.
